# Polio Survivors’ Experiences of Acute Phase Care After the Isolation Phase in the 1950s and 1960s in Finland

**DOI:** 10.1177/23333936241303730

**Published:** 2024-11-29

**Authors:** Minna Elomaa-Krapu, Marja Kaunonen

**Affiliations:** 1 Tampere University, Finland; 2 Metropolia University of Applied Sciences, Helsinki, Finland; 3 Wellbeing Services County of Pirkanmaa, Finland

**Keywords:** polio survivors, experiences, nursing, childhood, Finland

## Abstract

The aim of this study was to describe the childhood experiences of polio patients after the isolation phase of the disease in post-war Finland in the 1950s and 1960s. Qualitative empirical research was conducted. Interview material was gathered through theme-based written interviews, and the data were analyzed using reflective thematic analysis. The analysis resulted in the overarching main theme of “constructing psychological safety,” which reflected the themes “emotionally inadequate care,” “reclaiming physical identity,” and “the need to construct a child’s world.” These themes and their subthemes highlighted children’s experiences of the rules set by adults as well as their subordinate relationships with adults. Polio survivors’ experiences following the isolation phase of the disease were traumatic and demonstrated children’s inferior position in nursing in Finland in the 1950s and 1960s. Specifically, children recovering from polio experienced traumatic treatment and care and loneliness even beyond the isolation phase of the disease.

Polio is an RNA virus that spreads among populations via touch or droplet infection through the alimentary canal and into the bloodstream. Common symptoms include fever, headache, fatigue, stiffness in the neck or back, and pain in the limbs. Some infected persons remain asymptomatic. There is no cure for polio, but the symptoms can be treated. Vaccination is the most effective means of prevention (Khan, 2010; Oshinsky, 2005; Trevelyan et al., 2005). Polio has been detected in various Asian and African countries; the World Health Organization (WHO) is tracking these polio infections and continues to recommend inoculation (World Health Organization, 2021).

In the early 1940s, polio began to spread faster than ever before in North America and Europe, with cases peaking in the 1940s and 1950s (Alaranta et al., 2002; Altenbaugh, 2015; Muiluvuori, 2010; Nathanson & Kew, 2010; Rutty, 1996; Trevelyan et al., 2005). Polio vaccination campaigns began in Finland in 1957 (Muiluvuori, 2010). Roughly 700 to 800 polio cases were detected annually in Finland between years 1954 and 1956, and the overall number of all infected patients was estimated to be about 10,000 (Valtonen et al., 2005). Due to its national vaccination program, Finland was able to eradicate polio in the 1960s. However, in 1984, the disease reared its head again. The most recent case was reported in 1985, but further spread was prevented by a national vaccination campaign in the same year (Finnish Institute for Health and Welfare, 2019).

The onset of a polio infection was sudden, and the disease progressed in unusual ways, initially prompting speculation that it was caused by contaminated water and poverty (Carter, 2001; Highley, 2016). The symptoms were similar to those of other pediatric diseases, which made diagnosis challenging. The symptoms included a stiff neck, fever, nausea, and languor. Some infected individuals became paralyzed, and in others, the disease progressed to fatal respiratory failure. The difficulty of diagnosis and lack of knowledge about the origin and spread of the disease provoked fear among people (Carter, 2001; Highley, 2016; J. D. Wilson, 2005).

Children or teens diagnosed with polio in the 1900s had to isolate themselves immediately from their family and friends. Some acute-stage patients were isolated at home (Altenbaugh, 2015; Highley, 2016; Shell, 2005; J. D. Wilson, 2005). Isolation continued throughout the acute phase of the disease, which was when the risk of contagion was greatest. The disease was painful, as it damaged and destroyed nerve cells in the spinal column, and could even cause paralysis. The isolation stage lasted roughly two weeks but whole acute phase lasted all together approximately 4 weeks. Immediately afterward, patients began the tough rehabilitation phase in the hospital or at home. This could take from weeks to years, depending on the damage the virus had done to the patient’s body (Altenbaugh, 2015; Muiluvuori, 2010; J. D. Wilson, 2005). Patients experienced psychological symptoms as well as seizures that caused enormous levels of spinal pain to radiate throughout the body. Some suffered respiratory paralysis and were placed on ventilators, also called iron lungs. This treatment could last from a few days to several months, and for some, for the rest of their lives (Altenbaugh, 2015; Muiluvuori, 2010; J. D. Wilson, 2005). However, young polio survivors’ experiences of physical, psychological, and social suffering have not been acknowledged, and their childhood experiences have not been sufficiently examined from a nursing perspective globally (Altenbaugh, 2015; Highley, 2016).

Previous Finnish research has shown that survivors remember the isolation phase as a lonely and silent time in their lives. The presence of other pediatric patients with the same condition caused more fear than comfort. The treatment provided during the isolation period was perceived as cold and belittling. Children found their care to be neglectful, and they were not allowed to decide or be involved in their own care (Elomaa-Krapu & Kaunonen, 2024). These marked just the beginning of the painful treatments experienced by children during this period of loneliness.

However, the nurses involved in the treatment stated that they treated the children well; they were empathetic and took care of the children’s basic needs and cleanliness. The biggest challenge they faced was the lack of availability of necessary medication. In hospital settings, the sick drew strength from each other, helped each other as best they could, and played and read together. During visiting hours, they appeared to their parents to be brisk and happy. They hid their negative emotions. The culture of care and rehabilitation led them to portray themselves as strong children who would recover (Shell, 2005; J. D. Wilson, 2005).

Polio patients underwent traumatic experiences. During the diagnostic phase, children had to endure spinal taps to provide samples as well as other painful medical procedures. They were then isolated, during which time they enjoyed little human contact. After isolation, they were subjected to painful orthopedic and rehabilitation treatments. Plaster treatments, casts, and different physical therapies and braces all became familiar interventions that provided support and helped correct flexible deformities. Due to their illness, the patients’ bodies and physical autonomy were placed in the hands of others, adding to the children’s trauma (Altenbaugh, 2015; Highley, 2016). Children suffered pain, loss of self-identity and control, and social isolation from familiar surroundings and family. Feelings of insecurity led to a lack of trust in other people (Harrison & Stuifbergen, 2005).

In hospital settings, strict routines and limitations on the number of visitors allowed, even after the isolation period ended, exacerbated children’s sense of loneliness. Contact with their homes and parents was restricted because it was believed that youngsters would otherwise become disobedient. This caused for some young patients harder to recollect their siblings and homes because their hospital stays continued for years (Highley, 2016).

Previous study on nursing care for children’s polio, as given in Finnish nursing textbooks and nursing magazines, have shown that nurse-centered polio care was the practice in the 1950s and 1960s. Patient-centered nursing was still developing in Finland at the time, and although patients were treated kindly and respectfully, attitudes toward them were distant (Veltheim et al., 2024). According to J. D. Wilson (2005), patients were considered subordinate to nurses; therefore, nurses exhibited friendly but condescending attitudes toward patients. In the 1950s and 1960s, the presence of parents was not considered favorably in terms of a child’s care in a hospital, and nurses’ lack of empathy was evident in their attitudes toward children. Further, nurses were ignorant of children’s developmental, social, and psychological needs and were highly focused only on the child’s physical and medical needs. Overall, family-centered care (FCC) was faltering and slow, and the nursing culture was almost unchallengeable (Jolley & Shields, 2009).

The approach to children’s nursing has since advanced and is now based on the philosophy of FCC. It involves evaluating the positive impacts of families on the health and life outcomes of their children in hospital admissions. FCC emphasizes the relationship dynamics between a child patient and their parents, acknowledges the importance of maintaining a normal family life, and helps reduce the child’s stress (Kuo et al., 2012; Shields et al., 2012). FCC has been shown to improve patient care outcomes, increase families’ satisfaction with delivered care, raise families’ awareness of care needs, strengthen the parent–child bond, promote the effective use of health resources, and improve staff satisfaction (Çınar Özbay et al., 2023). The historical background of family-centered care encompasses the suffering and social changes that occurred after World War II. In the middle years of the 20th century, it was normal for parents worldwide to be unallowed to visit their children or to be allowed a visit of only half an hour per week (Jolley & Shields, 2009).

This research paper is part of a broader study aimed at describing the experiences of child polio patients and their siblings during the illness and treatment periods as well as the impact of the disease on entire families. This was the first nursing study to gather data on polio survivors’ experiences in Finland. The focus of the research was on post-war Finland in the 1950s and 1960s. The second aim of the study was to use the information gathered to enhance the literature on the history of care provided to children and their families, polio affliction and possible paralysis, and the importance of these experiences in Finnish society. The first part of the research, which dealt with the experiences of child polio survivors in the isolation phase, has already been published (Elomaa-Krapu & Kaunonen, 2024). The purpose of this article is to present the experiences of child polio patients after the isolation phase but before discharge or transfer to a rehabilitation hospital. The ultimate goal of this entire body of research is to demonstrate how child polio patients describe their experiences during the various stages of treatment, from falling ill to rehabilitation.

## Methods

### Aim

This study was conducted to determine the experiences of child polio patients during the post-isolation acute phase of their illness in Finnish hospitals in the 1950s and 60s. Post-isolation phase involved rehabilitation treatments, such as learning to move paralyzed limbs and walk again or other physical rehearsals. This paper does not consist of experiences after discharge or after the children were moved to other rehabilitation hospitals. The research question focused on how polio survivors described their experiences of the treatment and care they received in the post-isolation acute phase during their hospital stays.

### Theoretical Framework

A descriptive qualitative design was adopted for this study, with a primary emphasis on nursing research methodology and with the aim of providing an overall picture of the experiences of children affected by polio (Doyle et al., 2020). Data were collected through semi-structured thematic interviews (Qu & Dumay, 2011). The focus of the research was on understanding the subjective and collective emotions and experiences related to post-isolation events (Portelli, 1998). The process of recollecting and recounting memories was influenced by the roles and emotional states of the interviewees and interviewer and the trust built between the parties (Jones, 2004; Thompson, 2009). In research related to childhood memories, the moment of sharing experiences always involves two narrators: the adult relating the story and the child who reemerges through the recollection (Fass, 2010). The memories of polio survivors tend to be entwined with other aspects of life related to being a young person, an adult, and an elderly person. Memories, recollections, and retellings are thus tied to the life cycle of the research subject (Boschma et al., 2008; Thompson, 2009).

Childhood experiences are shaped by the prevailing societal and nursing culture. Therefore, childhood and childhood nursing care experiences reflect, on many levels, the expectations placed on child polio patients during different phases of the illness. Until the 1950s, polio patients in Finland were only treated in municipalities with infectious disease units or central hospitals. Meanwhile, rehabilitative or surgical treatments were available only in Helsinki (Muiluvuori, 2010). In the 1950s and 60s, people experienced life in agrarian post-war Finland and a time of hospital system reforms. There was a shortage of skilled nursing staff during this time (Leppo, 2013), so the care provided for polio patients may not have been uniform. Attitudes toward children changed after the wars and with an increase in the Finnish standard of living (Tuuteri, 1993). The creation of a safe atmosphere for care and education was the goal of childcare in post-war Finland (Malinen, 2013; Sihvonen, 2016).

This study was based on childhood theory (James et al., 1998) and trauma theory (American Psychiatric Association, 2000; Paivio & Pascual-Leone, 2023; van der Hart, 2021), centering the idea that children are socially constructed and belong to several social communities (James et al., 1998)., including their families, a post-war generation, an agricultural growth environment and its practices, school communities, and, eventually, hospitals and the nursing culture in Finland. The nursing theory in this research is based in previous research results that indicates the culture of nursing after the Second World War as well as the state of family-centered care. The experiences revealed in this study must be understood and positioned in the post-World War II era, acknowledging that nursing and medicine were just starting to develop and that behaviorism was the philosophy in child nursing from 1920 to 1970. Nurses were not ignorant, but they practiced the accepted thinking of the time period (Jolley & Shields, 2009).

### Design and Participants

This descriptive qualitative research was conducted to provide an overall picture of the experiences of children affected by polio (Doyle et al., 2020). A combination of thematic and dialogical interview methods was used in this study (Boschma et al., 2008; Shopes, 2011). The dialogical interview method involved asking participants to gather meaningful objects related to past events, including photographs, diaries, newspapers, magazines, and other memorabilia. During each interview, both the researcher and the participant looked at the gathered items that were meaningful to the participant; this facilitated easier recall of past experiences and triggered sensory memory (Denzin, 2001; Kvale, 2006; Tanggaard, 2009). The interview topics included background information about the participants and their families, childhoods, and family life before falling ill; hospital care during the acute and recovery stages; the return home; the impact of illness on the participants’ families; and family support.

A total of 49 people stricken with polio during childhood participated in this study. The interviews were conducted between September 29, 2018, and June 30, 2019. Forty-five of them were interviewed, and four provided written accounts of their experiences based on the research themes provided. The research participants included 32 women and 17 men, and their ages ranged from 66 to 80 years at the time of the interviews; the average age was 72 years. The age of one participant was missing from the accounts and was, therefore, not included in the calculation of the average age. Thirteen of the research subjects were under 3 years of age when they fell ill, while 15 were between 3 and 6 years of age. The ages of 17 participants ranged from 7 to 10 years when they first fell sick, two were between 11 and 14 years, and one was between 15 and 16 years. The onset age of one participant was not known, but it was estimated to be between 15 and 16 years based on the written background information. All interviews were conducted by the first author [M.E-K].

Thirty-nine participants recalled receiving hospital treatment during the acute phase of polio. Participants who were older than 3 years when they contracted the disease were able to describe their experiences with acute stage care, whereas those who were younger than 3 years described what they had been told by family members. The recollections of the latter were accepted for this study because they were similar to the narratives based on personal experiences.

The polio association helped in recruiting the study participants. The researcher called prospective participants and explained the nature of the study and the themes of the interview. Each participant received both oral and written information about the nature of the study. If the interviewees wished, a relative or next of kin could be present during the interviews. The interviews always ended with a discussion of the present and positive life events of the participants. The researcher verified the mental state of each participant and was prepared to refer the study participants to a healthcare practice if necessary. Each participant was allowed to talk about and share their experiences—a sign of mutual trust and empathy during the interviews (Strandèn, 2009).

### Data Analysis

Exploring experiences in healthcare requires the use of qualitative methods that involve meaningful, empathetic, and interpretive approaches. The goal is to understand and interpret experiences tied to social structures or time and place. The language used in the collected research material consequently becomes a tool that elicits such experiences. In the present study, the primary data—that is, participant interviews and transcriptions—were reviewed via reflective thematic analysis (Braun et al., 2022; Braun & Clarke, 2006, 2024; Clarke & Braun, 2020), which enabled the compilation of collective and subjective experiences. The research results were developed from and through data coding and only after reflective engagement. It was used to interpret the data as well as to uncover latent meanings after semantic readings. In a reflexive analysis, researchers place their own roles and experiences in active dialogs with the research material. Themes are interpretive stories about the data that constructed from the researcher’s subjectivity and are shaped through the accurate but situated reading of the data (Braun & Clarke, 2024).

### Ethical Considerations

This study complied with the Finnish National Board on Research Integrity guidelines for responsible scientific procedures and guidelines for studies with human participants and ethical reviews in Finland (Finnish National Board on Research Integrity, 2023). Relevant study permits were obtained for the study, and a preliminary ethical evaluation was requested from the Region Ethics Committee in 2018. The committee’s opinion was favorable, and they did not identify any ethical impediments in the study plan.

The study participants’ childhoods, illnesses, and treatment, nursing, and disability history as well as their impacts on the participants’ families were examined. Previously obtained information on the participants revealed that these child polio survivors felt powerless and demoralized. As a result, speaking about these experiences could cause feelings of vulnerability, psychological stress, and discomfort to arise if the participants’ memories triggered extremely traumatic experiences (Halbmayr, 2009). The interviewer observed the temperaments and stress levels of the study participants, offering breaks and the option to bypass questions that the participants did not want to answer. None of the participants felt unwell during the interviews and did not want to interrupt the interview.

### Trustworthiness

The traditional criteria for trustworthiness were used, namely credibility, transferability, dependability, and conformability (Lincoln & Guba, 1985). In this study, credibility was strengthened by creating a visual representation of the analysis flow and combining subordinate expressions with interpretations. However, it should be noted that in a reflexive thematic analysis, the researcher’s interpretation is a significant part of the meaning-making (Braun & Clarke, 2024). The results of this study cannot be directly transferred to other settings or groups, but they provide the possibility of understanding the potential experiences associated with similar illnesses. The transferability and authenticity of the research results were strengthened by accurate descriptions of the participants’ profiles, the context in which the experiences occurred, and direct quotes of the participants’ responses. Further, the researcher’s diary and observation notes during the interviews helped strengthen reliability and confirmability. The integrity of the analysis process was evaluated by another researcher as the process progressed.

## Results

**Figure 1.**
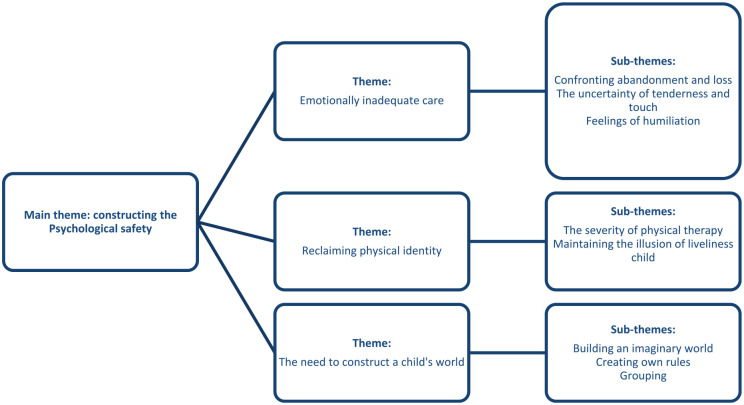
The main theme, secondary themes, and subthemes resulting from the thematic analysis.

The data analysis of the research material yielded the main theme of “constructing psychological safety,” which further reflected other experiences, such as “emotionally inadequate care,” “reclaiming the physical self,” and “the necessity of building a child’s world.” All the themes and subthemes mirrored children’s experiences of the rules of the adult world and their subordinate relationships with adults. For the study participants, the main theme of “constructing psychological safety” related to experiences of how child polio patients’ hopes and needs forced them in many different ways to create a sense of psychological safety on their own or with other children affected by polio. “To protect their identities and survive hospital stays, it was crucial for the children, either individually or collectively, to create their own worlds with their own rules. Grouping was inevitable, as the children also experienced abuse, and other children provided safety and protection. Figure 1 shows the themes and subthemes identified from the research results.

### Emotionally Inadequate Care

“Emotionally inadequate care” was a secondary theme that constructed from the participants’ experiences, and it consisted of experiences related to “confronting abandonment and loss,” “the uncertainty of tenderness and touch,” and “feelings of humiliation.” Confronting abandonment and loss was strongly reflected in experiences of rejection and fearfulness. Although the isolation phase had ended and the children were once more allowed to mingle with other patients, their sense of abandonment persisted. The connection with home had been severed, and feelings of homesickness endured. Some family members were not able to visit due to long distances, and patients who received visits from close relatives relived feelings of abandonment when the visits ended. Thus, the children repeatedly suffered feelings of rejection during their hospital stays. They felt abject alienation, even though other children were present, as paralysis meant that the afflicted children were unable to get physically closer to other young patients. The patients’ days revolved around hospital routines, and in their recollections, the participants stated that they often anticipated certain activities, even unpleasant ones, because they provided contact with adults. The child polio survivors’ experiences of loneliness were also tied to being minors. Some children had been placed in the same hospital ward as adults, which resulted in a sense of further isolation because they were invisible to adult patients. Their experiences also reflected how hospital staff would converse with and even divert adult patients but would ignore children. This underlined the children’s sense of abandonment and loneliness. The participants also described the recovery period, which they passed by waiting, playing solitary games, and engaging in imaginary mental play. Their days consisted of waiting for visitors, treatment routines, meal times, and changing times of day.

I don’t remember that, for example, the care staff would come and talk to me about [whether] I missed home or had any psychological problems. Or what I was possibly feeling while I was in [the] hospital. It probably wasn’t even part of the care personnel’s practice at that time either. There was this feeling of loneliness that stayed with me—that I was alone with this thing. Usually, my roommates were these older people, and two adult men, and obviously they talked with each other. But they didn’t take any special notice of me either, and they didn’t include me in their conversations. (Polio Survivor 6)

The feelings of abandonment and loss were also due to new losses experienced during the recuperation stage, which reinforced the sentiment that the children’s emotions and needs were not important. This experience was amplified when gifts or treats brought from home, which gave the young patients a sense of security, were handed over to other children or disappeared entirely. In this way, the patients’ ties to home were stolen. They also experienced being transferred to different wards or the relocation of their ward mates as new losses. The children’s fear experiences were multifaceted and included fear of the dark, hospital sounds, treatments, and the disease itself.

They weren’t all polio patients or children, but there were old people who were already at the end of [their] time. And there was no plan or what to do with me now. I just lay there. And I do remember that I slept with the old, dying man, and I was scared as hell that night. (Polio Survivor 15)

The term “poliomyelitis” inspired terror among the children because they did not understand what it meant, whether they would become completely paralyzed or at all, or if paralysis could worsen. These fears were also tied to concerns about suffocation, particularly among patients experiencing respiratory paralysis and those who were taken off their ventilators, either for hygiene routines or to practice breathing independently. In addition, the sounds of the breathing machines and the presence of children in worse conditions inspired terror.

Children experienced “the uncertainty of tenderness and touch,” as they received demonstrations of affection and physical contact only occasionally. Being treated to a smile or physical contact and being included in conversations added significantly to the children’s sense of security. Nurses were cast as substitute mothers, and doctors were viewed as father figures. The survivors’ longing for touch was evident in their recollections of bath time and hair washes, which were seen as pleasant moments that provided the contact they longed for. However, the research participants could not recall ever being held by a nurse.

I had a named nurse—I don’t even remember her name—but I always asked if she was on shift, and then it was always a moment of joy when she came, and I just sought that mother figure and mother’s protection from her. (Polio Survivor 12)

Although some children received smiles or attention, these moments were arbitrary and depended on individual staff members. The most prominent memories of the polio survivors included a sense of humiliation and being intimidated during treatment. This meant that the hospital staff abused their positions of power during the recovery phase and even resorted to mistreating the children. This was evident from the participants’ reports of being reprimanded by hospital staff if they could not properly perform physical tasks. They perceived the care provided as being cold and heavy-handed; they were told nothing, and their opinions were not solicited. This weakened the children’s confidence in adults. Some patients also felt that the nurses favored some children more than others and that their family backgrounds and the extent of their disability influenced how they were treated and the care they received. Being afflicted by the disease was seen as the result of sins committed by the children or their families. Nurses also frightened the children by declaring that the devil would take them away or that they would not go to heaven when they died.

Of course we were intimidated with talk that Lucifer would come and that we would be taken by some horned creature. We didn’t even really understand what that was, but we knew that it was something bad and scary. It’s really unpleasant to talk about all this because I also want to understand the nurses’ position. There were also nurses who drew caricatures of us in a notebook, and then they put the images on the table when I’d been naughty, and that was incredibly embarrassing for us kids. I was so ashamed because of that book, and I was afraid that it would be brought out. (Polio Survivor 43)

### Reclamation of Physical Identities

The polio survivors experienced a powerful breakdown and reconstruction of their physical selves during their childhoods. The rehabilitation of their paralyzed bodies during various training sessions stood out in their daily routines. However, these experiences highlighted “the severity of physical therapy” and the approach of “maintaining the illusion of liveliness” as a result of the research analysis. The treatment methods were described as harsh and experimental. Patients with paralyzed lower limbs were made to stand to see whether they could remain upright; when the children fell, the nurses made no attempt to catch them. On a daily basis, patients with respiratory paralysis were forced to see how long they could last without the help of a ventilator. This practice caused them to lose consciousness and experience the sensation of suffocation. The children’s experiences included forcible straightening of the limbs and being made to sit up; none of the treatments were explained to them in advance.

So this nurse still proudly tells the doctor that this girl is so cured, so healthy, that now you show the senior doctor how well you can already be up. And when I got out of bed, I fell on the floor, so I didn’t have any strength in my legs. And I started to cry terribly, and it was such a big setback for me mentally that it was as if I had messed up. (Polio Survivor 24)

The young patients were also scolded for soiling or wetting themselves, even if they were paralyzed or could not walk or stand upright. They felt responsible for their infirmity and, as a result, experienced guilt and embarrassment because of their disability. At the beginning of the rehabilitation phase, the use of a harness to help with walking was compared to walking a dog, and this, combined with the children’s disability, caused a powerful sense of shame. They also experienced embarrassment when having to use a rectal thermometer in a large open room and when they were made to walk naked in a corridor while a doctor assessed their gait and posture. The children did not understand why they had to walk naked in front of others, and they experienced physical as well as emotional humiliation. They were also subjected to harsh punishments. For instance, a paralyzed child who was considered disobedient would be turned to face the wall or shut in a dark bathroom. This intensified the child’s feelings of shame, inadequacy, and objectification.

And then there was this corridor [for] walking, and you always had to be almost naked. People were coming and going there, [and] some relative might even turn up. It definitely wasn’t any kind of intimate space. So it was kind of, it felt like being in front of some kind of panel. It reminded me of some kind of beauty pageant; it was all the same to me. And you’d be there, and there would be comments like, “Yes, they’re still dragging that leg. No, they’re not lifting it.” That kind of constant criticism while you were walking there, and then the staircases, and I remember thinking that I would never walk up the stairs. (Polio Survivor 18)

The study participants experienced their rehabilitation as if it were to be achieved on command, and, as children, they felt embarrassed if they were unable to do as instructed. They felt that they had to maintain the illusion of a lively child. This meant that the children soon learned to project vitality when interacting with nursing staff and family members. During rehabilitation sessions, the nursing staff encouraged the children to be positive. The children did not want to disappoint their family members; they felt the need to inspire joy in their relatives and pretend to be upbeat recovering patients for fear of being reprimanded by the hospital staff. The study subjects recalled rehabilitation as mainly learning to walk again. Other child patients also upheld the young polio patients’ motivation to walk again so that they could talk to each other. Some research participants stated that the exercise sessions were uplifting, while others saw them as inescapable.

There was a girl opposite there, a girl who probably waved at me. I slowly learned to walk over there to the girl’s bed and eventually I got there. That it was that kind of rehabilitation. (Polio Survivor 4)

### The Importance of Constructing a Child’s World

The research analysis indicated that to survive their harsh and emotionally bereft treatment, it was necessary for the children to create their own worlds. The construction of a child’s world consisted of “building an imaginary world,” “creating own rules,” and “grouping.” This was evident from the safe worlds and stories that the children concocted, which often focused on being discharged from the hospital or running away. The children drew comfort from building their own worlds. For example, they imagined that they would heal by Thursday or that they could listen and dance to music. Imaginary worlds were important because they allowed the children to exist in the adult world.

I then came up with this story that my father is a train driver, and my father is coming home from the evening shift today to pick me up. Yes, at first, those roommates were a bit of a believer, but it doesn’t mean you’re lying. (Polio Survivor 12)

The children also created their own rules. This was crucial, as it helped offset the hospital’s strict rules and routines. The children climbed out of bed as their range of mobility improved; they crawled on the floor, some escaped to other rooms or floors in the hospital, and some even let animals in through the window. Breaking adult rules made the children momentarily happy. They felt compelled to experience something other than the routines created by adults, if only for a minute. The importance of creating their own worlds was evident from the fact that any event that deviated from hospital conventions, including holidays such as Easter and Christmas and new experiences such as a nurse making a tent for the children in the hospital ward, brought comfort to the young patients. Creating imaginary worlds and rules gave the children a fleeting sense of security.

We were mean. We figured out that when you have a mirror in your hand and the sun shines on it, it makes rays of light on the ceiling and then we started telling each other ghost stories and spinning the mirror, after which the patient, who was younger than us, was so scared. (Polio Survivor 42)

Grouping was a consequence of the lack of empathy and harsh discipline the children endured. They needed to defend and look after the well-being of other children, and it was easier to contend with the adult world as a group. The children sought out others in different rooms or wards and crawled toward other patients. It was also easier to break rules as a group. They decided to hide food in bed linen or in furniture together. At times, the formation of groups manifested negatively, as the patients teased other children. Coming together as a group also facilitated the creation of shared imaginary worlds, which sometimes included escape plans.

So we climbed over each other in bed and did this and that—you know how kids are. And we’d crawl along the floor, and once, I even managed to go so far. I remember that I went into a cleaning closet, and I had a kind of silent protest. Once, I went up the stairs on hands and knees to an adult ward, and, of course, I was caught right away. “What are you doing here?” And I was sent back immediately, and I was so happy that I’d managed to pull off such a prank. (Polio Survivor 18)

## Discussion

The results of this study indicate that the research participants underwent traumatic treatment-related experiences that continued beyond the isolation phase (Elomaa-Krapu & Kaunonen, 2024). The findings highlighted the necessity for the child polio patients to create their own worlds and rules because they were invisible to adults. In the adult-dominated hospital environment, the children had no voice, and their feelings were not acknowledged. We can only speculate whether their invisibility was due to the fundamental status of children in general or of children with disabilities in particular. The children suffered both emotional and physical abuse due to the experimental nature of the treatment. It was also both intentional and unintentional. The intentional nature was tied to the patients’ identities as children living with disabilities as well as the treatment they received. This resulted in ridicule, harsh discipline, and force-feeding. Inadvertent abuse included the pain and fear caused by experimental treatments, such as standing and walking exercises, baths, and ventilator weaning. Previous research has revealed that the treatment of polio patients was callous in the 1940s and 1950s; no emotional support was provided, and their ties to home were not nurtured (Schanke et al., 1999).

In this study, the subtheme “the illusion of enthusiasm” meant that the children adapted to appear unrelentingly upbeat. Disability was undesirable, and the children soon learned to please others and do everything in their power to ensure that the nursing staff and their families could celebrate their rehabilitation (Schanke et al., 1999). Studies from the 1940s and 1950s have shown that children learn to deliberately appear happy and optimistic. In the present study, the children’s emotional care that they received in acute phase of the care, led the patients feeling that they were to blame for their illness and disability. This finding is supported by research conducted by Schanke et al. (1999). Further, the illusion of cheerfulness supported the view that child polio survivors had to learn to walk again, although this wasn’t always realistic (D. J. Wilson, 2008). This viewpoint arose from the perspectives of disability culture that prevailed during those times. Specifically, disability was seen as repulsive and a source of shame. People living with disabilities were hidden and shut away from society and had to be made “healthy” and fit for society as soon as possible (Rogers, 2014).

This study showed that during the acute phase of the disease, the children’s daily routines were determined by hospital rules and adult norms. The accounts of the polio survivors strongly reflected children’s insignificance in the eyes of adults. No one asked about them or about their needs and hopes. Placing these children in the same room as others reinforced their sense of loneliness and invisibility. They were deprived of a child’s world and, at the same time, were not allowed into the adult world. Yoshida and Shanouda (2015) reported that children living with disabilities have very little control over their bodies and treatment. We surmise that this lack of control had a significant impact on the children’s tendency to form groups. They attempted to break rules and sought companionship and comfort from each other. This study also clearly demonstrated the children’s need to construct their own version of reality, with their imaginations serving as coping mechanisms.

The Finnish hospital system was modernized in the 1950s and 1960s (Vauhkonen, 1992) and was just beginning to develop children’s medical care after the Second World War. Children were initially treated alongside adults and did not have any special status in hospital care (Tuuteri, 1993). This was vividly reflected in the narratives shared by the study participants. Although children’s well-being became topical in the 1950s and Finland had begun to set targets for family care (Malinen, 2013; Sihvonen, 2016), this was not mirrored in the study participants’ experiences. There was also a shortage of nursing skills in Finland at the time. Finland’s post-war status in the 1940s and 1950s further hindered the skill development of nursing personnel (Muiluvuori, 2010).

The study findings showed that children were reprimanded, intimidated, and punished for misbehaving. The most traumatic approach to discipline involved shutting children in a dark room. In early theoretical writings, children and childhood have been depicted as either good or evil, and children had to be raised and molded into decent citizens (James et al., 1998). At the 1950s and 1960s in Finnish culture, the use of sanctions was a part of children’s upbringing and education. Negative sanctions included scolding, intimidation, threats, and even corporal punishment (Aukia, 2010). Based on the study results, it can be concluded that nursing personnel also followed this culture of reward and punishment in the 1950s and 60s.

The study also uncovered the practice of having children walk naked in hospital hallways so that the staff could assess their physical rehabilitation. The children found these activities embarrassing. In early 20th-century Finland, children’s health was evaluated using physical metrics, and these measurements were gathered through social demographic programs. For example, it was completely normal to see a queue of undressed children during school examinations (Tuomaala, 2004). It appears that children recovering from polio were objectified targets of measurement and treatment.

The focus of this study was on the theories of childhood described by James et al. (1998). The polio survivors’ narratives highlighted the hospital norms, adult rules, and concepts of education that prevailed in those decades. These meant that children were treated and cared for to ensure they would become obedient and good-humored patients, in line with the culture at the time. Children were not perceived as visible or active participants in their own care. In the hospital setting, children recovering from polio formed groups akin to secret societies, which reinforced the feeling that they were, above all, children with their own needs. They formed close bonds, defended each other, and shared care experiences as well as brutal rehabilitation and treatment experiences. According to James et al. (1998), we can speak of the “minority children’s group.” when we talk about polio children and their experiences in Finland. In the present study, this relates to the inability of doctors and nurses to understand children’s needs and misery, exemplified by the fact that the children were given the same behavioral demands as adult patients. This was also caused by the nursing culture at a time when nurses’ competence with family-centered care had just started to develop globally (Jolley & Shields, 2009). In Finland, the perception of healthcare changed to focus on families and children after the Second World War. The concepts of home, core family, and motherhood were highlighted in society. Further, the importance of healthcare and prevention was acknowledged, and child health clinics were developed and improved (Harjula, 2007).

### Strengths and Limitations of the Study

While interviews provide valuable research material, it must be noted that people tend to recount what they believe to be worthy of sharing. In this study, 20 of the 49 research subjects recalled the hospital recuperation phase of their illness, and the results reflect their stories. However, it cannot be assumed that the results apply to everyone who was infected with or disabled by polio. The research material also revealed the collective experiences of children who contracted polio. The purpose of the study was not to create a generalized oral history but to highlight children’s experiences of illness and treatment. Nevertheless, research into past experiences and emotions can complement the general history of nursing, treatment, and medicine.

The study participants described events that took place decades ago. As a result, it was difficult for them to recall some experiences, and some events might have been remembered inaccurately. In general, those who were 3 years old at the time of their illness retained some memories, while those who were older recalled more. When reporting childhood events, it is not possible to say for certain whether the narratives were based on the narrator’s own memory or learned from others over the years. The recording of memories might also have been affected by the suppression of painful or traumatic childhood events as part of the body’s defense mechanism. Further, translations of the accounts may lack the linguistic richness of the original expressions. These are all factors that should be considered when evaluating the research results.

For the interviews, the study participants were asked to bring memorabilia from the time they were ill or hospitalized. Given the era considered in this study, not all participants had photographs, and some had lost their items from that time. During the course of the interviews, the researchers reviewed the available items, including photos, toys, and treatment records. These items played an important role in activating the participants’ memories. During the analysis, the researchers actively entered their own thinking into dialog with the research data, and the results were developed through interpretation.

This research provided information about past treatment experiences with historic and social significance in Finland, and not all of it was positive. This research report was created in compliance with responsible scientific practices and is not aimed at criticizing past events (Boschma et al., 2008) but at considering those experiences in combination with the prevailing culture of care and education and the level of nursing staff competence.

## Conclusion

Although polio is no longer a global threat to human health, international mobility still contributes to the transmission of infectious diseases. At the same time, vaccine hesitancy has become more widespread, leading to unvaccinated children. This study provides insights into disease, care, and rehabilitation experiences that can be referred to when treating other injured or seriously ill patients.

We conclude that in the 1950s and 1960s, the collective experiences of child polio patients in the hospital during the recovery phase were traumatic and placed the children in a subordinate position in Finnish nursing care. No previous research has been conducted on the acute phase experiences of child polio survivors in Finland. Therefore, this study holds social, cultural, and historical significance, addressing the lack of knowledge on the subject. The polio survivors’ experiences of the illness and treatment process affected their quality of life and their roles in society. Information about these experiences is important, particularly from the perspective of nursing history, because people living with permanent disabilities and patients subjected to experimental treatments during the 1950s and 1960s have the right to be heard when receiving care and treatment. There is currently no comprehensive information on this subject in Finland, which makes the present study important.

The study participants’ experiences of feeling neglected, lonely, misheard, and abused led to trauma and feeling shame. Being shut off in their own imaginations was important for coping with traumatic experiences. The construction of imaginary worlds was a result of trauma and a way for maltreated children to adapt to painful and frightening experiences. The study findings showed that the children suffered from pain, a loss of identity and self-determination, and social alienation from familiar environments and their families. Therefore, there is a need to inspect past approaches in Finnish nursing culture and consider the negative aspects of nursing culture to widen the understanding of Finland’s nursing history.

In sum, the major impacts/contributions of this study are threefold. The study findings add to our understanding of the history of care for children and families and, more specifically, for those sickened and disabled by polio, promoting our understanding of the importance of their experiences in Finnish society and healthcare settings. The study revealed that care during the acute phase involved neglecting children and that emotional support was random. The findings also provide insights into the modern approach to providing care for children suffering from infectious diseases and foster an appreciation of patients who are still suffering from post-polio symptoms.

In the future, it is necessary for researchers to study patients’ experiences during the rehabilitation phase as well as during surgical care and after returning home and readapting to society. It is also necessary to study possible support structures for the families of people living with disabilities in Finland.
